# Recombination at the emergence of the pathogenic rabbit haemorrhagic disease virus *Lagovirus europaeus*/GI.2

**DOI:** 10.1038/s41598-020-71303-4

**Published:** 2020-09-02

**Authors:** Joana Abrantes, Clément Droillard, Ana M. Lopes, Evelyne Lemaitre, Pierrick Lucas, Yannick Blanchard, Stéphane Marchandeau, Pedro J. Esteves, Ghislaine Le Gall-Reculé

**Affiliations:** 1 CIBIO/InBio-UP, Centro de Investigação em Biodiversidade e Recursos Genéticos, Research Network in Biodiversity and Evolutionary Biology, Universidade do Porto, Campus de Vairão, Rua Padre Armando Quintas, 4485-661 Vairão, Portugal; 2 Departamento de Biologia, Faculdade de Ciências da Universidade do Porto, Porto, Portugal; 3 Unité de Virologie, Immunologie, Parasitologie, Aviaires et Cunicoles, Laboratoire de Ploufragan-Plouzané-Niort, Agence nationale de sécurité sanitaire, de l’alimentation, de l’environnement et du travail (Anses), Ploufragan, France; 4 Instituto de Ciências Biomédicas Abel Salazar (ICBAS)/Unidade Multidisciplinar de Investigação Biomédica (UMIB), Universidade do Porto, Porto, Portugal; 5 Unité de Génétique Virale et Biosécurité, Laboratoire de Ploufragan-Plouzané-Niort, Agence nationale de sécurité sanitaire, de l’alimentation, de l’environnement et du travail (Anses), Ploufragan, France; 6 Unité Petite Faune Sédentaire et Espèces Outre-Mer, Direction de la Recherche et de l’Appui Scientifique, Office Français de la Biodiversité (OFB), Nantes, France

**Keywords:** Viral evolution, Molecular evolution

## Abstract

Rabbit haemorrhagic disease is a viral disease that emerged in the 1980s and causes high mortality and morbidity in the European rabbit (*Oryctolagus cuniculus*). In 2010, a new genotype of the rabbit haemorrhagic disease virus emerged and replaced the former circulating *Lagovirus europaeus*/GI.1 strains. Several recombination events have been reported for the new genotype *Lagovirus europaeus*/GI.2, with pathogenic (variants GI.1a and GI.1b) and benign (genotype GI.4) strains that served as donors for the non-structural part while GI.2 composed the structural part; another recombination event has also been described at the p16/p23 junction involving GI.4 strains. In this study, we analysed new complete coding sequences of four benign GI.3 strains and four GI.2 strains. Phylogenetic and recombination detection analyses revealed that the first GI.2 strains, considered as non-recombinant, resulted from a recombination event between GI.3 and GI.2, with GI.3 as the major donor for the non-structural part and GI.2 for the structural part. Our results indicate that recombination contributed to the emergence, persistence and dissemination of GI.2 as a pathogenic form and that all described GI.2 strains so far are the product of recombination. This highlights the need to study full-genomic sequences of lagoviruses to understand their emergence and evolution.

## Introduction

Since the 1980s, European rabbits worldwide, either domestic or wild, have been affected by rabbit haemorrhagic disease^1^. This highly contagious and fatal disease is caused by the rabbit haemorrhagic disease virus (RHDV), a single-stranded positive-sense RNA virus that belongs to the family *Caliciviridae*, genus *Lagovirus*. Benign rabbit caliciviruses that confer more or less protection against pathogenic strains, as well as moderately pathogenic strains, including Michigan rabbit calicivirus (MRCV), have also been described^2–7^.

In 2010, a new pathogenic lagovirus was identified in France^8^, formerly designated as RHDV2 or RHDVb and now as *Lagovirus europaeus*/GI.2 according to a proposal for a unified nomenclature for lagoviruses^9^. GI.2 caused unusual mortalities in rabbits vaccinated against GI.1 (former G1-G6) strains^8, 10^. Further analysis revealed genetic differences that were reflected in its positioning in a phylogenetic tree based on capsid protein (VP60) sequences. GI.2 constituted a new phylogenetic group^8, 11^ with more than 15% divergence from all know benign and pathogenic lagoviruses. Other unique characteristics include its ability to fatally infect rabbits younger than two months (previously resistant to the disease)^11, 12^ and several hare species (*Lepus* spp.)^13–17^. Differences in disease duration, mortality rates, and in the frequency of occurrence of subacute/chronic forms have also been documented^11^; moreover, since 2016, virulence of some GI.2 strains has increased to reach that of GI.1 viruses^18^. As for the other lagoviruses, the origin of GI.2 is still unknown.

Following its detection in France, GI.2 rapidly migrated through Europe, but also reached more distant places such as Oceania, Africa, and North America^19^, revealing an efficient dispersal and establishment. Replacement of the former circulating GI.1 strains was also reported^11, 20–23^. In the Iberian Peninsula, emergence of GI.2 was accompanied by several recombination events^24, 25^. These events involved the pathogenic GI.1b variant that circulated only in Iberian rabbit populations, and benign GI.4 strains (RCV-A1). In Australia, a recombinant GI.1a/GI.2 strain was recently characterised in rabbit and hare samples^26^. A recurrent recombination breakpoint was found at the boundary of the non-structural and structural encoding genes (between RdRp and VP60). A second recombination breakpoint was further detected at the junction of the two genes encoding the non-structural proteins p16 and p23^25^. While the biological implications of the different genomic composition of the recombinant strains remains to be assessed, it is clear that recombination plays a central role in the evolution of GI.2. Recombination might have also precipitated the emergence of GI.1 as a pathogenic form^27^, although this still remains under debate. Indeed, two hypotheses had been suggested for the emergence of pathogenic lagoviruses: either from a pre-existing benign lagovirus or following a species jump^28, 29^. Both hypotheses are sustained by multiple pieces of evidence and are not mutually exclusive, but they require further confirmation, especially following the recent emergence of the new pathogenic genotype GI.2.

In this study, and in order to disclose the emergence of pathogenic rabbit haemorrhagic disease virus *Lagovirus europaeus*/GI.2, we sequenced the full-length genomic sequences of two GI.2 strains collected in the earliest outbreaks^11^ and the coding sequences of two GI.2 strains collected more recently. We obtained also four new full coding sequences of benign GI.3 (RCV-E1) strains collected in France between late 2007 and early 2009, since GI.3 appears to have a closer relationship with GI.2^30^. Maximum-likelihood (ML) analyses were performed considering the recombination breakpoint identified by recombination detection methods^24, 25^.

## Results

In this study, we obtained eight new genome sequences: four for GI.3 and four for GI.2. These included four strains previously identified based on their capsid sequences as GI.3 (JA10/08-10, BO25/08-133, JA34/09-48 and CHA20/09-100; GenBank accession numbers: LT708119, LT708122, LT708127 and LT708128, respectively) and two strains as GI.2 (10-28 and 10-32; GenBank accession numbers: HE800531 and HE800532, respectively)^9, 11^. We obtained the full-length genomic sequences of strains 10-28 and 10-32, the complete coding sequences (CDS) of strains 16-35 and 16-36, and the CDS along with the 3′ untranslated region (UTR) of the strains JA10/08-10, BO25/08-133, JA34/09-48 and CHA20/09-100.

The genomes of the earliest GI.2 strains 10-28 and 10-32 were 7,448 nucleotides (nt) long and shared 94.6% nucleotide identity. The length of the 5′ UTR sequences is  9 nt and that of the 3′ UTR is 70 nt. The closest strains were GI.3 strains with an average nucleotide identity of  93.8% (94.3% for the non-structural part of the genomes), while the average identity with GI.1 strains was 85% (86.4% for the non-structural part of the genomes). The genomes of the GI.3 strains were 7,427 nt long (excluding the 5′ UTR), except the CHA20/09-100 strain that was 7,424 nt long. The 3′ UTR sequences had the same length (64 nt). When including the GI.3 06-11 sequence (GenBank accession number MN737115), the average nucleotide identity between the five GI.3 strains was 97.1%. The CDS of the four GI.2 showed between 93.2% and 94.5% of nucleotide identity (93.2% between 16-35 and 16-36 strains), and when compared to GI.1 CDS presented no deletions or insertions (7,369 nt long), while the GI.3 CDS presented a deletion of six consecutive nucleotides within the capsid gene (7,363 nt long instead of 7,369 nt). This deletion is present in the benign strains 06-11, Ashington and the Italian RCV (GenBank accession numbers: EF558587 and X96868)^2, 4, 7^ and comprises a codon putatively under positive selection in GI.1 strains^31^. The strain CHA20/09-100 had a further three nucleotide deletion in the minor structural protein VP10 (positions 193–195, amino acid 65).

**Figure 1:**
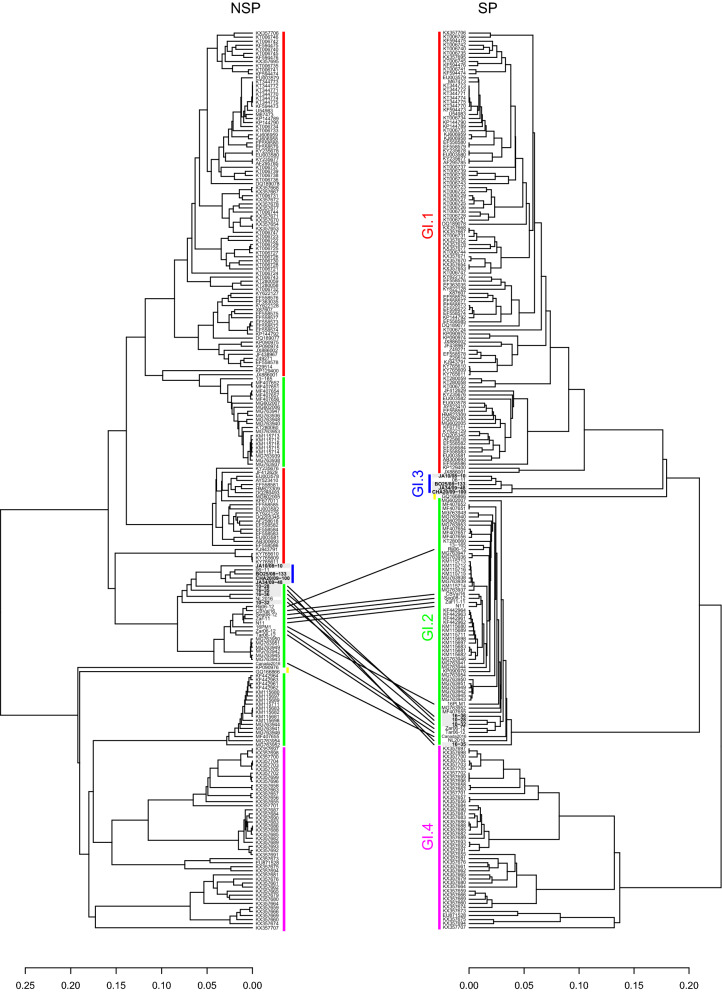
Tanglegram for the non-structural (NSP) and structural genes (SP) of the 221 lagoviruses sequences. For the tanglegram for the non-structural genes (NSP), colouring of the sequences is according to the genotype to which the strains belong to for the structural genes (SP): GI.1 (red); GI.2 (green); GI.3 (blue); GI.4 (purple); MRCV (yellow). Lines highlight the recombinant GI.3/GI.2 strains that were identified in the RDP recombination analysis. Sequences obtained in this study appear boxed in grey.

The complete dataset (221 sequences; 7,368 nucleotides) was screened for recombination with RDP (Recombination Detection Program)^32^. The different methods available in RDP detected as recombinants, with strong statistical support (p-values < 0.05; Table 1), all the strains from the early GI.2 outbreaks (strains 10-28, 10-32 and N11), all the GI.2 strains previously identified as non-recombinants (strains CBVal16, Zar11-11, Rij06-12, Tar06-12, Zar06-12 and Seg08-12) and GI.2 strains more recently detected (16PLM1, NL2016, Canada2016, 16-35, 16-36). A single recombination breakpoint was determined at the non-structural/structural parts boundary (Table 1). For most of these strains, the GI.3 strain 06-11 was identified as the most likely donor for the non-structural part, with the exception of strain 16-36 for which the most likely donor is CHA20/09-100. A GI.2 strain (7-13_Barrancos_Portugal_2013 or SOS155_Portugal_2015, GenBank accession numbers KF442963 and MG763946, respectively) was the most likely donor for the structural part. Thus, according to RDP all these sequences (GI.2 strains from the early outbreaks and GI.2 non-recombinant strains) are GI.3/GI.2 recombinants. The tanglegram based on the alignments for the non-structural and the structural genome partitions also evidenced as recombinants the strains identified by RDP (Fig. 1).

**Table 1:** Results of the RDP recombination analysis.

Strains	Recombination breakpoint (nucleotide positions)a	Most likely donor strain		Methods and average p-value						
		Non-structural proteins	Structural proteins	RDP	GENECONV	BootScan	MaxChi	Chimaera	SiScan	3Seq
10-28 10-32 N11	5,242–5,319	06-11 (GI.3; accession number MN737115)	7-13 Barrancos (GI.2; accession number KF442963)	1.061 × 10^–126^	8.713 × 10^–70^	6.006 × 10^–125^	2.539 × 10^–27^	9.056 × 10^–36^	1.075 × 10^–44^	1.604 × 10^–10^
CBVal16 Zar11-11 Tar06-12 Zar06-12 Rij06-12 Seg08-12										
16PLM1 NL2016 Canada2016										
16-35										
16-36	5,231–5,423	CHA20/09-100 (GI.3; accession number LT708128)	SOS155 (GI.2; accession number MG763946)	2.991 × 10^–108^	2.061 × 10^–91^	**–**	1.196 × 10^–32^	3.575 × 10^–32^	3.432 × 10^–39^	3.401 × 10^–10^

^a^99% confidence interval.

**Figure 2:**
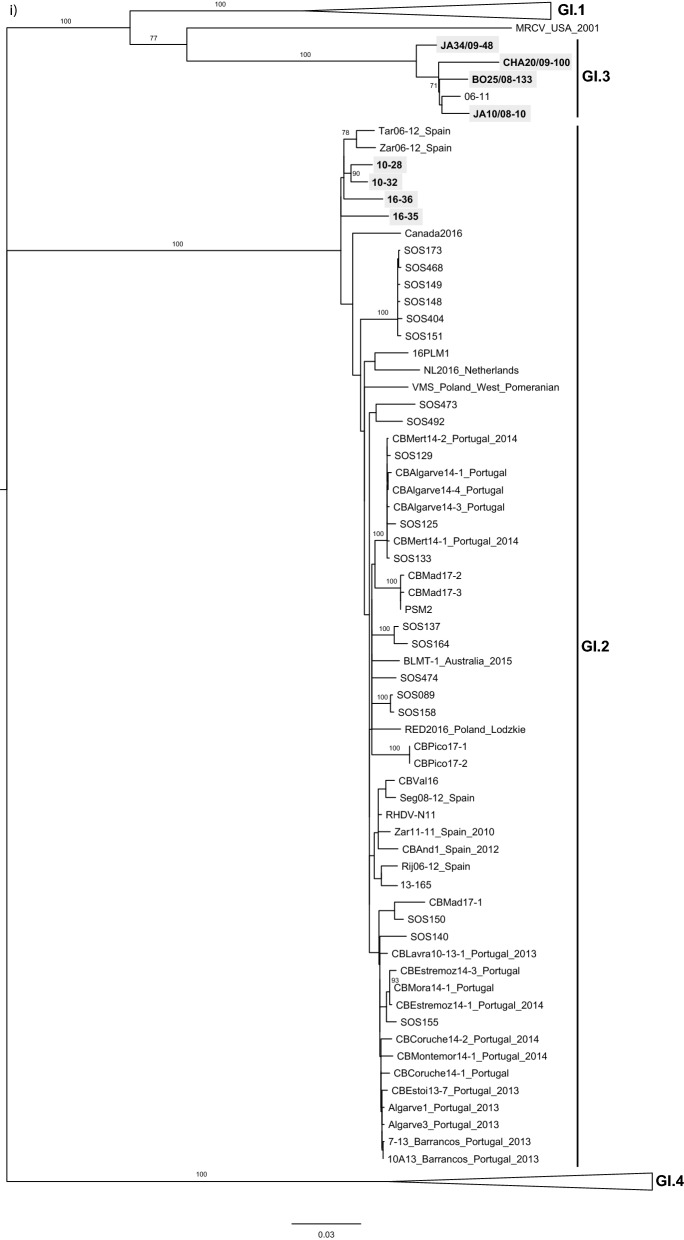

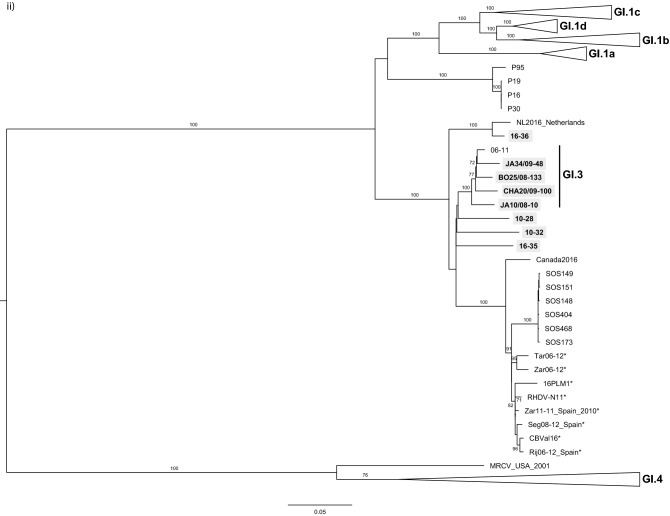
Maximum Likelihood (ML) phylogenetic trees for (**i**) the structural genes VP60 + VP10 (n = 221 sequences; nucleotides 5,296–7,369; nucleotide substitution model GTR + G + Γ_4_), and (**ii**) the non-structural genes except p16 (n = 221 sequences; nucleotides 430–5,295; nucleotide substitution model GTR + G + Γ_4_). Horizontal branch lengths are drawn to scale of nucleotide substitutions per site and the trees are mid-point rooted. The percentage of trees in which the associated taxa clustered together was determined from 1,000 bootstrap replicates and is shown next to the branches (only bootstrap values ≥ 70 are shown). Genotype or variant assignment is according to the ML tree for the structural genes. Sequences obtained in this study appear boxed in grey. *indicates strains considered previously non-recombinant GI.2, but now identified as GI.3/GI.2 recombinants. GenBank accession numbers of the sequences used are listed in supplementary information.

The phylogenetic analysis was performed taking into account the location of the recombination breakpoint detected by RDP at the junction of the non-structural and structural parts. In the ML tree of the structural part, which includes VP60 and VP10 (Fig. 2i), the GI.3 strains formed a strongly supported group (bootstrap value 100), that was a sister group to GI.1 (bootstrap value 100). The MRCV strain, a GI.4-like/GI.3-like recombinant^33^, also grouped with the GI.3 strains (bootstrap value 77). According to the nomenclature recently proposed for lagoviruses, MRCV does not meet the criteria to be considered as a member of the GI.3 group and is currently unclassified^9^. Indeed, the genetic distance between MRCV and GI.3 VP60 gene sequences is > 15% (data not shown) and no other similar strains (from independent outbreaks) had been identified. As for the four GI.2 sequences obtained in this study, they clustered within a highly supported group (bootstrap value 100) formed by all the strains previously identified as having a GI.2 capsid, and that is more closely related to GI.1 and GI.3 than to GI.4. Two strains collected in the Netherlands^34^ and Canada in 2016 and that were recently made available (GenBank accession numbers MN061492 and KY235675, respectively) were also part of the GI.2 cluster.

Regarding the ML tree of the non-structural part except p16 (Fig. 2ii), the four GI.2 strains obtained in this study 10-28, 10-32, 16-35 and 16-36 clustered with the GI.3 strains. This highly supported cluster (bootstrap value 100) further includes strains SOS148, SOS149, SOS151, SOS173, SOS404 and SOS468 previously identified as GI.4/GI.2/GI.2 (GI.4 for p16, and GI.2 for the remaining genome regions)^25^, GI.2 strains previously considered as non-recombinants (marked with an *), and the 2016 strains from Canada (Canada2016) and the Netherlands (NL2016).

The ML tree for the non-structural protein p16 (see supplementary information), recapitulated most of the results observed in the ML tree for the non-structural part with the exception of the positioning of the Portuguese strains SOS137, SOS164, SOS148, SOS149, SOS151, SOS173, SOS404 and SOS468, that appear clustered with GI.4. Strains SOS137 and SOS164 were previously shown to be triple recombinants between GI.4, GI.1b and GI.2 (GI.4 for p16, GI.1b for the remaining non-structural proteins and GI.2 for the structural proteins)^25^ which is in agreement with the results obtained in this study. As for strains SOS148, SOS149, SOS151, SOS173, SOS404 and SOS468, the tree suggests that their genome also originated from three different strains: GI.4 for p16, GI.3 for the remaining non-structural proteins and GI.2 for the structural proteins. In this case, it most likely involved recombination between a GI.4 strain and a GI.3/GI.2 recombinant strain. A tanglegram analysis and RDP further confirmed these results (see supplementary information).

## Discussion

GI.2 was identified as a novel pathogenic form of lagovirus in France in 2010^8^. Field observations and further characterisation of the strains revealed unique characteristics in comparison with former strains such as the ability to cross the species boundaries^13–17^ and to kill young rabbits^11, 12^. Previous studies also revealed the occurrence of recombination of GI.2 strains with non-pathogenic (GI.4) and pathogenic (GI.1a and GI.1b) strains^24–26^, showing an important role of recombination in generating diversity in GI.2 and confirming the high capacity of recombination within lagoviruses.

Our results show that the strains circulating at the time of the first noticed GI.2 outbreaks were already recombinants between a European non-pathogenic strain (GI.3), that was donor for the non-structural part, and the new lagovirus genotype, GI.2, that formed the structural part. Indeed, in our ML tree for the non-structural part, the strains from the first GI.2 outbreaks (10-28 and 10-32) grouped with the non-pathogenic GI.3 group, while for the structural part these strains clustered with all the strains of the newly emerged GI.2 genotype. Strains from the earliest outbreaks in Spain and Portugal in 2011–2012^24^, and strains collected in the Canary Islands (Spain)^35^, France, the Netherlands^34^ and Canada in 2016 are also GI.3/GI.2 recombinants. These results, that were confirmed by the RDP, the ML trees and the tanglegram analyses, demonstrate that all the GI.2 strains described so far, including those that were considered non-recombinants and that in some instances co-circulated with other recombinant GI.2 strains^24^, resulted from recombination. Several recombination events are thus associated with the evolution of this new genotype: with GI.3, GI.4, GI.1b and GI.1a, with a recombination breakpoint at the non-structural/structural boundary^24, 26^. An additional recombination event was further previously reported with a breakpoint at the p16/p23 boundary and involved GI.4 as the donor of p16, while GI.3/GI.2 (see also supplementary information) or GI.1b/GI.2 recombinants were the donors for the remaining viral genome^25^.

Notably, the pattern described here for lagoviruses, with a recombination hotspot at the start of the major structural gene that encodes the capsid, is typical of caliciviruses^36–38^. Indeed, the combination of low sequence divergence^39^, presence of complex RNA secondary structures^39^ that may promote template switching by the virus polymerase, and the existence of a subgenomic RNA^40^ that may act as a secondary template when RNA replication is resumed upon template switching, seem to contribute for the high rates of recombination observed in this region in the genome of caliciviruses and that likely constitutes an important survival strategy in the evolution of this family^41^. This strategy, that resembles antigenic shift in Influenza virus^41^, allows caliciviruses to rapidly generate diversity by producing new genomic combinations, which might be beneficial for the adaptation to new hosts and environments, and to overcome selective pressures. Recombination is important in shaping the evolution of RNA viruses, including the closely related picornaviruses^42^ and coronaviruses^43^ and, more importantly, is often associated with the emergence of new viruses^44^ and even families, e.g. the *Hepeviridae* family^45^. As for the recombination involving the breakpoint at the p16/p23 boundary, it may have originated from a non-replicative recombination mechanism where RNA strands are randomly self-ligated or joined by cellular ligases^46^. Atypical recombination breakpoints such as this have been also observed for other caliciviruses and a similar mechanism proposed^47^.

Estimation of the time to the most recent common ancestor (tMRCA) of GI.2 by using capsid sequences pointed to an emergence in July 2008^25^. Although the estimated substitution rates may be inaccurate due to limited sampling, reduced sequence variation and low temporal spread, they tend to underestimate rather than overestimate the real tMRCA^48, 49^. Thus, it is possible that despite surveillance efforts, GI.2 circulated unnoticed in wildlife prior to its detection^50^. The virus that recombined with GI.3 to produce this new genotype is currently unknown and undetected, even with the molecular surveys of lagoviruses performed in European leporids and our improved knowledge of the complete coding sequences of both pathogenic and benign lagoviruses. The same occurs for some older recombinant Iberian strains where the virus that originated the non-structural part has never been detected^51^. Both viruses, the one originating GI.2 and the one of these Iberian strains, could have either circulated harmlessly prior to their detection, thus making difficult to detect them, or have become extinct due to their lower fitness as a non-recombinant form, as suggested for noroviruses that recombined and whose partial sequences were never found^52^.

The evidence for recombination with GI.1a^26^, GI.1b^24^, GI.3 and GI.4^24, 25^ (see also supplementary information) reveals that GI.2 successfully recombines with a great diversity of pathogenic and non-pathogenic lagoviruses. This, together with the tMRCA estimates and the hypothesis of circulating harmlessly before its emergence, supports an evolution from a non-pathogenic form that acquired pathogenicity^28^. We suggest that evolution of pathogenicity was not driven solely by point mutations, but was aided by recombination events. Similarly to other RNA viruses, lagoviruses genomes may function as interchangeable modules rather than as strict genomes, which leads to the appearance of mosaic-like genomes through recombination, resulting in a semi-independent evolution of structural and non-structural genes^53, 54^ and with low-fitness regions being eliminated by recombination^55^. This might help to explain how a non-pathogenic strain (GI.3), combined with a potentially benign strain (GI.2), gave rise to a highly lethal, pathogenic strain. Non-pathogenic forms are thus likely to be involved in the evolution of pathogenic forms and recombination might have precipitated the emergence of *Lagovirus europaeus*/GI.2. The hypothesis of a species jump may not be discarded. Indeed, reservoir species could have acted as source for the original virus that jumped to the final host, the European rabbit^28^. Whether recombination occurred in the reservoir species or in the final host is unknown. Nonetheless, reservoir species have yet to be identified in which lagoviruses could have evolved and replicated without being detected before “jumping” into the European rabbit (Le Gall-Reculé, personal communication)^56–59^. Genetic mechanisms such as recombination require co-infection of a host cell by two (or more) viruses. The high frequency of recombination detected in lagoviruses also implies a high frequency of co-infection, either in the host population or in the reservoir species. Leeks and colleagues established a link between co-infection and viral diversity, as more diverse populations can produce more virulent infections and better adapt to new hosts^60^. This might explain the array of hare species infected by GI.2^13–17^, which constitutes a novelty in relation to GI.1.

Our results add complexity to the diversity of lagoviruses, but help to elucidate our current understanding on the emergence of pathogenic forms. In addition, they show that full genomic sequences of lagoviruses are crucial to understand their evolutionary history and genetic relationships. Future studies should focus on unravelling the role of the structural and the non-structural parts in the emergence of pathogenicity in lagoviruses.

## Methods

### Virus samples and full-length genome sequencing

No animals were captured, handled or killed specifically for the purpose of this study. Duodenum samples were collected from four hunted French rabbits by the National Hunting and Wildlife Agency (ONCFS) during the hunting seasons in 2007–2008 and 2008–2009. Presence of lagoviruses had previously been detected in these samples and phylogenetic relationships of the obtained capsid VP60 gene sequences had been determined^9^; these were identified as GI.3 strains. Four liver samples were further collected in France from dead rabbits collected in three rabbitries affected by RHD, two on November 2010 (10-28 and 10-32 GI.2 strains^11^) and one in June 2016 (strain 16-35), and in the field on June 2016 (strain 16-36). The presence of GI.2 in the 16-35 sample was diagnosed by Labovet Conseil (Les Herbiers, France). We characterised this strain as well as the 16-36 one based on their complete VP60 gene sequences, confirming that they belong to the GI.2 genotype.

For the sequencing of the complete coding genome of GI.3 strains, ~ 30 mg of duodenal tissue were homogenized in a mixer-mill disruptor (TissueLysed, Qiagen, Hidden, Germany). RNAs were first extracted with TRIzol^®^ (TRIzol LS Reagent, Ambion, Thermo Fisher Scientific, Villebon-sur-Yvette, France) before using the RNeasy Mini kit (Qiagen, Hilden, Germany) to increase sensitivity^2^. For GI.2 strains, RNA was extracted from 100µL of liver exudate using the RNeasy Mini kit. Except for two GI.2 strains (16-35 and 16-36) for which the coding sequence was obtained using NGS (see below) from purified and quantified RNA using the RNeasy MinElute cleanup kit (Qiagen, Hilden, Germany) and the Qubit RNA HS Assay Kit with the Qubit 2.0 Fluorometer (Thermo Fisher Scientific, Villebon-sur-Yvette, France), respectively, RNAs were reverse-transcribed using oligo-dT as a primer and Maxima Reverse Transcriptase (Thermo Fisher Scientific, Villebon-sur-Yvette, France). Protocols were performed as recommended by the manufacturers.

According to the strains, different PCR strategies were attempted for genome sequencing either by using combinations of newly designed primers (primer sequences available upon request) or primers previously published^3, 11, 14^. Thus, cDNAs of JA10/08-10, BO25/08-133 and CHA20/09-100 GI.3 strains were amplified using several overlapping PCRs, including one that covered the recombinant breakpoint between the non-structural and structural encoding genes, using Expand High-fidelity PCR System (Roche, Sigma-Aldrich, Saint-Quentin-Fallavier, France). For JA34/09-48 GI.3 strain, due to lower viral loads, after two first PCRs that amplified two overlapping fragments of about 5,700 and 6,700 bp, respectively, several nested or semi-nested overlapping PCRs were performed. The complete coding sequences of the four GI.2 genomes were obtained following either one PCR amplification of about 7,300 bp using the primers 1U^14^ and 15L^11^ followed by smaller overlapping PCRs for sequence confirmation (10-28 and 10-32; primer sequences available upon request), or NGS (16-35 and 16-36, see below). For these two last strains, the 5′ end sequence of the CDS (530 bp) was confirmed using the PCR primers 1U and 1L as described in Le Gall-Reculé et al. (2017)^14^. PCRs were performed using Expand High Fidelity enzyme (Roche-Applied-Science). The different PCR products were analysed by electrophoresis, purified using the MinElute PCR Purification kit (Qiagen, Hilden, Germany) and quantified using the Qubit dsDNA HS Assay Kit with the Qubit 2.0 Fluorometer (Thermo Fisher Scientific, Villebon-sur-Yvette, France). DNA sequences were determined with an ABI Prism 3100 Genetic or a 3500 Series Genetic Analysers in both directions (Applied Biosystems, Foster City, CA, USA), using the PCR and several inner primers and Big Dye Terminator v3.1 (Life Technologies) as recommended by the manufacturer. The 5′ and 3′ ends of the 10-28 and 10-32 strains were obtained using the rapid amplification of cDNA ends (RACE) method following the protocol developed in Lemaitre et al. (2018)^30^. PCR products were purified and sequenced as described above. Consensus sequences were compiled using Vector NTI Advance software (Life Technologies, Thermo Fisher Scientific, Villebon-sur-Yvette, France).

For NGS, cDNA libraries were prepared using the Ion Total RNA-Seq Kit (Life Technologies, Carlsbad, CA, USA) according to a protocol adapted from the supplier’s instructions (available upon request from the authors). Sequencing was performed using Ion Proton Sequencer (Life Technologies). The reads were cleaned with the Trimmomatic^61^ (version 0.36) software (ILLUMINACLIP: oligos.fasta: 2:30:5:1: true; LEADING: 3; TRAILING: 3; MAXINFO: 40:0.2; MINLEN: 36). Then a Bowtie2^62^ (version 2.2.5) alignment was performed with cleaned reads on local nucleotide databank to identify lagovirus references. All cleaned reads aligned by BWA^63^ (version 0.7.15-r1140) on "*Oryctolagus cuniculus*" (reference genome form EnsEMBL) were excluded from further analysis. The references with the highest number of matching reads were used for a new alignment with BWA-MEM. The aligned reads of this third alignment were collected and then down-sampled to fit a global coverage depth estimation of 80x. These cleaned reads were submitted to the SPAdes^64^ (version 3.10.0) de novo assembler and their raw related reads to the Mira^65^ (version 4.0.2) de novo assembler. The *de novo* contigs were then submitted to MegaBLAST^66^ on a local copy of nucleotide databank. The best lagovirus reference identified with MegaBLAST was used in a last BWA alignment of cleaned reads.

The genomic sequences produced in this study have been deposited in GenBank under the accession numbers: MN746288, MN746289, MN737116, MN737117 (GI.3 strains JA10/08-10, BO25/08-133, JA34/09-48 and CHA20/09-100, respectively); MN737113, MN737114, MN738377, MN786321 (GI.2 strains 10-28, 10-32, 16-35 and 16-36, respectively).

### Recombination analysis

Complete coding sequences obtained in this study were aligned with publicly available complete coding sequences of *Lagovirus europaeus* representing genotypes GI.1, GI.2, GI.3 and GI.4 in BioEdit software version 7.0.3^67^. The final dataset included 221 sequences, 7,369 nucleotides in length (see supplementary information for the list of the sequences used). The dataset was screened for recombination by seven methods available in the RDP software version 4.4 (RDP, GENECONV, BootScan, MaxChi, Chimaera, SiScan and 3Seq)^32^ with the following parameters: sequences were set to linear, Bonferroni correction, highest acceptable p-value of 0.05 and 500 permutations. Only recombination events detected by three or more methods were considered.

Recombinant strains were visualized by plotting a tanglegram using the “dendextend package”^68^ in the RStudio software (version 3.6.1)^69^.

### Phylogenetic analysis

Pairwise nucleotide distance comparison (p-distance model) was computed using MEGA6^70^.

Following the results obtained with RDP, phylogenetic analyses were carried out separately for the following genome partitions: nucleotide positions (i) 1–429 (p16; supplementary information), (ii) 430–5,295 (p23, p37, p29, VPg, protease and RdRp), and (iii) 5,296–7,369 (VP60 and VP10). Maximum-likelihood phylogenetic trees were inferred in MEGA6^70^ using the best model of nucleotide substitution determined in the same software for the different genome partitions, according to the lowest AICc value (Akaike information criterion, corrected). Support for each cluster was obtained from 1,000 bootstrap replicates.

## Supplementary information

Supplementary Information.
