# Supplementary material for: Recombination at the emergence of the pathogenic rabbit haemorrhagic disease virus Lagovirus europaeus/GI.2
Source: Sci Rep. 2020 Sep 2;10:14502. doi: 10.1038/s41598-020-71303-4 (PMC7468141; doi:10.1038/s41598-020-71303-4)
Supplement: Supplementary file 1 — Supplementary Information. [file 41598_2020_71303_MOESM1_ESM.pdf]

**Supplementary Information**

**Authors:** Joana Abrantes, Clément Droillard, Ana M. Lopes, Evelyne Lemaitre, Pierrick Lucas, Yannick Blanchard, Stéphane Marchandeau, Pedro J. Esteves, Ghislaine Le Gall-Reculé

**Title:** Recombination at the emergence of the pathogenic rabbit haemorrhagic disease virus *Lagovirus europaeus*/GI.2

Table. List of the 221 sequences used in the study and the genotype/variant they belong to according to their VP60.

| GenBank accession number | Strain name | Genotype/Variant (VP60) |
|---|---|---|
| AB300693 | Hokkaido/2002/JPN_2002 | GI.1a |
| AF258618 | Iowa2000_USA | GI.1a |
| AF295785 | Mexico89_1989 | GI.1c |
| AY523410 | CD/China | GI.1a |
| DQ189077 | Bahrain_2001 | GI.1d |
| DQ189078 | Saudi_Arabia | GI.1c |
| DQ205345 | JX/CHA/97_China_1997 | GI.1a |
| DQ280493 | ChinaWHNRH | GI.1a |
| EF363035 | clonepJG-RHDV-DD06_Ramsay Island_1999 | GI.1d |
| EF558572 | Frankfurt12_Germany_1996 | GI.1d |
| EF558573 | Frankfurt5_Germany | GI.1d |
| EF558574 | Wika_Germany_1996 | GI.1d |
| EF558575 | Ascot_UK_1992 | GI.1d |
| EF558576 | Jena_Germany | GI.1d |
| EF558577 | Meiningen_Germany_1993 | GI.1d |
| EF558578 | Eisenhuttenstadt_Germany_1989 | GI.1b |
| EF558579 | NZ54_New Zealand_2003 | GI.1c |
| EF558580 | NZ61_New Zealand_2003 | GI.1c |
| EF558581 | Erfurt_Germany_2000 | GI.1a |
| EF558582 | Dachswald_Germany_2000 | GI.1a |
| EF558583 | Triptis_Germany_1996 | GI.1a |
| EF558584 | Rossi_Germany_2002 | GI.1a |
| EF558585 | Hagenow_Germany_1990 | GI.1a |
| EF558586 | Hartmannsdorf_Germany_1989 | GI.1a |
| EU003578 | IN-05_USA_2005 | GI.1a |
| EU003579 | Italy90_1990 | GI.1c |
| EU003580 | Korea90_1990 | GI.1c |
| EU003581 | NY-01_2001 | GI.1a |
| EU003582 | UT-01_2001 | GI.1a |
| EU871528 | RCV-A1_Australia_2007 | GI.4 |
| GQ166866 | MRCV_USA_2001 | GI.3 |
| HM623309 | NJ-2009_China_2009 | GI.1a |
| JF412629 | HYD_China_Heilongjiang_2005 | GI.1a |
| JF438967 | CB156_Portugal_1997 | GI.1b |
| JX886001 | CB194_Portugal_2006 | GI.1b |

| | | |
|---|---|---|
| JX886002 | CB137_Portugal_1995 | GI.1b |
| KF442961 | Algarve1_Portugal_2013 | GI.2 |
| KF442962 | Algarve3_Portugal_2013 | GI.2 |
| KF442963 | 7-13Barrancos_Portugal_2013 | GI.2 |
| KF442964 | 10A13Barrancos_Portugal_2013 | GI.2 |
| KF594473 | Czechoslovakia-V351 1987 | GI.1c |
| KF594474 | Turretfield_06_Australia_2006 | GI.1c |
| KF594475 | Turretfield_07_Australia_2007 | GI.1c |
| KF594476 | Turretfield_09_Australia_2009 | GI.1c |
| KF677011 | STR2012_Poland/2012 | GI.1a |
| KJ606958 | Gungahlin_Australia_1998 | GI.1c |
| KJ606959 | Parkwood_Australia_1998 | GI.1c |
| KJ943791 | P95_Portugal_1996 | GI.1b |
| KM115680 | CBEstoi13-7_Portugal_2013 | GI.2 |
| KM115681 | CBEstremoz14-1_Portugal_2014 | GI.2 |
| KM115682 | CBEstremoz14-3_Portugal | GI.2 |
| KM115683 | CBMora14-1_Portugal | GI.2 |
| KM115689 | CBMontemor14-1_Portugal_2014 | GI.2 |
| KM115697 | CBCoruche14-1_Portugal | GI.2 |
| KM115698 | CBCoruche14-2_Portugal_2014 | GI.2 |
| KM115711 | CBLavra10-13-1_Portugal_2013 | GI.2 |
| KM115712 | CBMert14-1_Portugal_2014 | GI.2 |
| KM115713 | CBMert14-2_Portugal_2014 | GI.2 |
| KM115714 | CBAlgarve14-1_Portugal | GI.2 |
| KM115715 | CBAlgarve14-3_Portugal | GI.2 |
| KM115716 | CBAlgarve14-4_Portugal | GI.2 |
| KM878681 | N11_Spain_2011 | GI.2 |
| KM979445 | CBVal16_Portugal_2012 | GI.2 |
| KP090974 | CB110_Portugal_1994 | GI.1b |
| KP090975 | CB154_Portugal_1997 | GI.1b |
| KP090976 | CBAnd1_Spain_2012 | GI.2 |
| KP129395 | Rij06-12_Spain | GI.2 |
| KP129396 | Seg08-12_Spain | GI.2 |
| KP129397 | Tar06-12_Spain | GI.2 |
| KP129398 | Zar11-11_Spain_2010 | GI.2 |
| KP129399 | Zar06-12_Spain | GI.2 |
| KP129400 | Bar01-11_Spain_2011 | GI.1b |
| KP144789 | Pd_Poland/1989 | GI.1c |
| KP144790 | KGM_Poland/1988 | GI.1c |
| KP144792 | BLA_Poland/1994 | GI.1d |
| KT006721 | NZL/Canterbury/Lincoln/517/2013 | GI.1c |
| KT006722 | NZL/Canterbury/MackenzieBasin/95/2012 | GI.1c |
| KT006723 | NZL/HawkesBay/BoundaryStream/94/2012 | GI.1c |
| KT006724 | NZL/Canterbury/Hawarden/320/2013 | GI.1c |
| KT006725 | NZL/Otago/Queensberry/74/2013 | GI.1c |
| KT006726 | NZL/Otago/Taras/61/2014 | GI.1c |
| KT006727 | NZL/Otago/Luggate/67/2014 | GI.1c |
| KT006728 | NZL/Otago/Alexandra/64/2014 | GI.1c |
| KT006729 | NZL/Otago/Wanaka/63/2014 | GI.1c |
| KT006730 | NZL/Canterbury/MackenzieBasin/322/2013 | GI.1c |
| KT006731 | AUS/NSW/Hall/WAT1-13/2007 | GI.1c |
| KT006732 | AUS/NSW/Murrumbateman/BlueGums/2014 | GI.1c |
| KT006733 | AUS/WA/Gnowangerup/1999 | GI.1c |

| | | |
|---|---|---|
| KT006734 | AUS/SA/FlindersRanges/1999 | GI.1c |
| KT006735 | AUS/WA/Bunbury/2000 | GI.1c |
| KT006736 | AUS/SA/Robe/2000 | GI.1c |
| KT006737 | AUS/SA/FlindersRanges/2002 | GI.1c |
| KT006738 | AUS/SA/OneTreeHill/2003 | GI.1c |
| KT006739 | AUS/SA/Coorong/2004 | GI.1c |
| KT006740 | AUS/SA/BillaKallina/2004 | GI.1c |
| KT006741 | AUS/SA/Woodcroft/2005 | GI.1c |
| KT006742 | AUS/SA/FlindersRanges/2006 | GI.1c |
| KT006743 | AUS/TAS/SouthArm/2007 | GI.1c |
| KT006744 | AUS/SA/Hillbank/2008 | GI.1c |
| KT006745 | AUS/SA/Bulgania/2009 | GI.1c |
| KT006746 | AUS/WA/WonganHills/2012 | GI.1c |
| KT006747 | AUS/NSW/Grafton/2013 | GI.1c |
| KT280058 | BlueGums-2_Australia_2015 | GI.1c |
| KT280059 | BLMT-2_Australia_2015 | GI.1c |
| KT280060 | BLMT-1_Australia_2015 | GI.2 |
| KT344770 | AUS/CSIRO/Master | GI.1c |
| KT344771 | AUS/CSIRO/Working | GI.1c |
| KT344772 | AUS/CSIRO/Release | GI.1c |
| KT344773 | AUS/EMAI/Batch-1A | GI.1c |
| KT344774 | AUS/EMAI/Batch-1D | GI.1c |
| KT344775 | NZL/V351/Control | GI.1c |
| KX357653 | AUS/ACT/AIN-1/2009 | GI.1c |
| KX357654 | AUS/ACT/AIN-2/2009 | GI.1c |
| KX357655 | AUS/ACT/Gudg-26/2013 | GI.4 |
| KX357656 | AUS/ACT/Gudg-79/2014 | GI.4 |
| KX357657 | AUS/ACT/Gudg-89/2014 | GI.4 |
| KX357658 | AUS/ACT/GUN_1-240/2012 | GI.4 |
| KX357659 | AUS/ACT/GUN_1-29/2008 | GI.4 |
| KX357660 | AUS/ACT/GUN-121/2010 | GI.4 |
| KX357661 | AUS/ACT/GUN-159/2011 | GI.4 |
| KX357662 | AUS/ACT/GUN-160/2011 | GI.4 |
| KX357663 | AUS/ACT/GUN-238/2012 | GI.4 |
| KX357664 | AUS/ACT/GUN-343/2014 | GI.4 |
| KX357665 | AUS/ACT/GUN1-11/2007 | GI.4 |
| KX357666 | AUS/ACT/GUN1-22/2008 | GI.4 |
| KX357667 | AUS/ACT/GUN1-37/2009 | GI.1c |
| KX357668 | AUS/ACT/GUN1-52/2009 | GI.1c |
| KX357669 | AUS/ACT/GUN1-60/2010 | GI.4 |
| KX357670 | AUS/ACT/MtPt-2/2010 | GI.1c |
| KX357671 | AUS/ACT/MtPt-4/2010 | GI.1c |
| KX357672 | AUS/ACT/PI-1/2009 | GI.1c |
| KX357673 | AUS/NSW/BUR1-1/2007 | GI.4 |
| KX357674 | AUS/NSW/CAT_2-12/2007 | GI.4 |
| KX357675 | AUS/NSW/CAT_3-4/2007 | GI.4 |
| KX357676 | AUS/NSW/LWR-1/2010 | GI.4 |
| KX357677 | AUS/NSW/M2/2007 | GI.1c |
| KX357678 | AUS/NSW/M9/2007 | GI.1c |
| KX357679 | AUS/NSW/MIC3-3/2007 | GI.4 |
| KX357680 | AUS/NSW/MIC5-8/2007 | GI.4 |
| KX357681 | AUS/NSW/OAK_NT-12/2011 | GI.4 |
| KX357682 | AUS/NSW/OC-13/2007 | GI.4 |

| | | |
|---|---|---|
| KX357683 | AUS/NSW/OC-15/2007 | GI.4 |
| KX357684 | AUS/NSW/OC-18/2007 | GI.4 |
| KX357685 | AUS/NSW/OC-20/2007 | GI.4 |
| KX357686 | AUS/NSW/OC-26/2007 | GI.4 |
| KX357687 | AUS/NSW/OC-33/2007 | GI.4 |
| KX357688 | AUS/NSW/OC-36/2007 | GI.4 |
| KX357689 | AUS/NSW/OC-39/2007 | GI.4 |
| KX357690 | AUS/NSW/OC-7/2007 | GI.4 |
| KX357691 | AUS/NSW/V-4/2007 | GI.4 |
| KX357692 | AUS/NSW/V-5/2007 | GI.4 |
| KX357693 | AUS/NSW/V-8/2007 | GI.4 |
| KX357694 | AUS/NSW/WAU-1/2009 | GI.4 |
| KX357695 | AUS/SA/ORA383/2008 | GI.1c |
| KX357696 | AUS/VIC/BEN-10/2009 | GI.4 |
| KX357697 | AUS/VIC/BEN-115/2010 | GI.4 |
| KX357698 | AUS/VIC/BEN-124/2010 | GI.4 |
| KX357699 | AUS/VIC/BEN-35/2009 | GI.4 |
| KX357700 | AUS/VIC/BEN-52/2010 | GI.4 |
| KX357701 | AUS/VIC/BM-2/2007 | GI.4 |
| KX357702 | AUS/VIC/BM-35/2009 | GI.4 |
| KX357703 | AUS/VIC/BM-45/2009 | GI.4 |
| KX357704 | AUS/VIC/BM-49/2009 | GI.4 |
| KX357705 | AUS/VIC/BM-60/2009 | GI.4 |
| KX357706 | AUS/WA/B.Hill/2013 | GI.1c |
| KX357707 | NZ/Southland/Gore-425A/2013 | GI.4 |
| KY235675 | QC/Canada/WIN-AH-2016-OTH-0018_2016 | GI.2 |
| KY235676 | MB/Canada/WIN-AH-2011-OTH-026_2011 | GI.1a |
| KY235677 | Mexico/2718_Mexico | GI.1c |
| KY235678 | Korea/2719_Korea | GI.1c |
| KY622127 | P158_Portugal_1998 | GI.1d |
| KY622128 | P165_Portugal_1998 | GI.1d |
| KY622129 | P175_Portugal_1999 | GI.1a |
| KY765609 | P16_Portugal_1994 | GI.1b |
| KY765610 | P19_Portugal_1994 | GI.1b |
| KY765611 | P30_Portugal_1994/1995 | GI.1b |
| LT168844 | 13-165_France_2013 | GI.2 |
| M67473 | FRG_Germany_1989 | GI.1c |
| MF407651 | CBPico17-1_Portugal_2017 | GI.2 |
| MF407652 | CBPico17-2_Portugal_2017 | GI.2 |
| MF407653 | 16PLM1_Spain_2016 | GI.2 |
| MF407654 | PSM2_Portugal_2016 | GI.2 |
| MF407655 | CBMad17-1_Portugal_2017 | GI.2 |
| MF407656 | CBMad17-2_Portugal_2017 | GI.2 |
| MF407657 | CBMad17-3_Portugal_2017 | GI.2 |
| MG602005 | BBI_Poland_Mazowieckie | GI.1a |
| MG602006 | RED2016_Poland_Lodzkie | GI.2 |
| MG602007 | VMS_Poland_West Pomeranian | GI.2 |
| MG763936 | SOS089_Portugal_2014 | GI.2 |
| MG763937 | SOS125_Portugal_2014 | GI.2 |
| MG763938 | SOS129_Portugal_2014 | GI.2 |
| MG763939 | SOS133_Portugal_2014 | GI.2 |
| MG763940 | SOS137_Portugal_2015 | GI.2 |
| MG763941 | SOS140_Portugal_2015 | GI.2 |

| | | |
|---|---|---|
| MG763942 | SOS148_Portugal_2015 | GI.2 |
| MG763943 | SOS149_Portugal_2015 | GI.2 |
| MG763944 | SOS150_Portugal_2015 | GI.2 |
| MG763945 | SOS151_Portugal_2015 | GI.2 |
| MG763946 | SOS155_Portugal_2015 | GI.2 |
| MG763947 | SOS158_Portugal_2015 | GI.2 |
| MG763948 | SOS164_Portugal_2015 | GI.2 |
| MG763949 | SOS173_Portugal_2015 | GI.2 |
| MG763950 | SOS404_Portugal_2015 | GI.2 |
| MG763951 | SOS468_Portugal_2015 | GI.2 |
| MG763952 | SOS473_Portugal_2015 | GI.2 |
| MG763953 | SOS474_Portugal_2015 | GI.2 |
| MG763954 | SOS492_Portugal_2016 | GI.2 |
| MN061492 | NL2016_Netherlands | GI.2 |
| U54983 | RHDV-V351_1987 | GI.1c |
| X87607 | BS89_Italy_1989 | GI.1d |
| Z29514 | SD_France_1989_ | GI.1b |
| Z49271 | RHDV-AST89_1989 | GI.1b |
| MN737113[*] | 10-28_France_2010 | GI.2 |
| MN737114[*] | 10-32_France_2010 | GI.2 |
| MN737115 | 06-11_France_2006 | GI.3 |
| MN737116[*] | JA34/09-48_France_2009 | GI.3 |
| MN737117[*] | CHA20/09-100_France_2009 | GI.3 |
| MN746289[*] | BO25/08-133_France_2008 | GI.3 |
| MN746288[*] | JA10/08-10_France_2008 | GI.3 |
| MN738377[*] | 16-35_France_2016 | GI.2 |
| MN786321[*] | 16-36_France_2016 | GI.2 |

[*]strains obtained in this study.

Figure. Tanglegram for p16 and non-structural genes except p16 (NSP-p16) of the 221 lagoviruses sequences. Colouring of the sequences is according to the genotype to which the strains belong to for the structural genes: GI.1 (red); GI.2 (green); GI.3 (blue); GI.4 (purple); MRCV (yellow). Lines highlight the recombinants at the p16/p23 boundary. Sequences obtained in this study appear in bold.

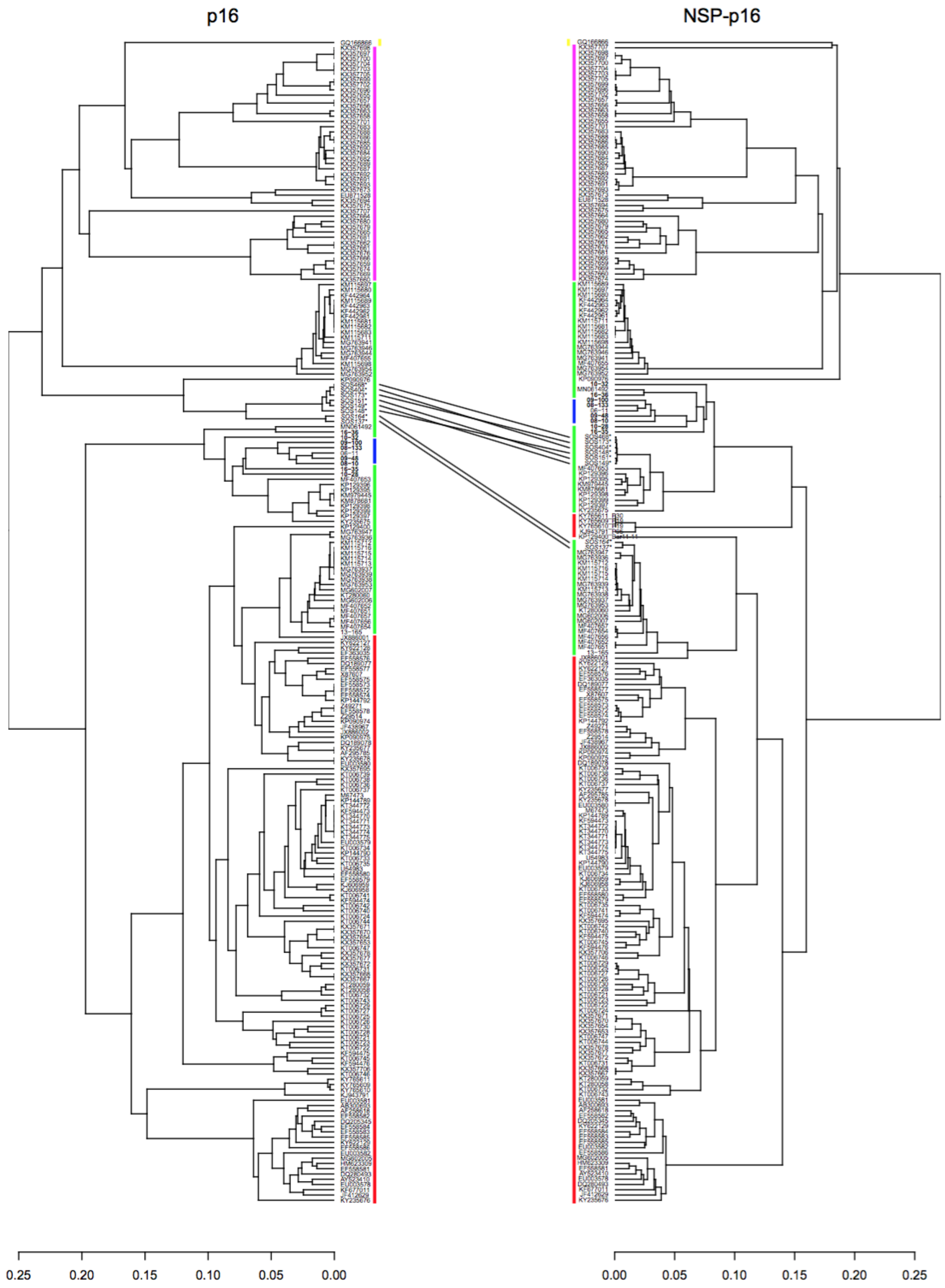

Table. Results of the RDP recombination analysis at the p16/p23 boundary.

| | | Most likely donor strain | | Methods and average p-value | | | | | | |
|---|---|---|---|---|---|---|---|---|---|---|
| Strains | Recombination breakpoint (nucleotide positions)[a] | p16 | Non-structural proteins | RDP | GENECONV | BootScan | MaxChi | Chimaera | SiScan | 3Seq |
| SOS137, SOS164 | Undetermined-369 (355-374) | SOS173 (GI.4) | CBAlgarve14-3 (GI.1b) | $2.417x10^{-34}$ | $1.219x10^{-28}$ | $7.549x10^{-34}$ | $5.432x10^{-8}$ | $5.017x10^{-8}$ | $2.925x10^{-7}$ | $1.489x10^{-18}$ |
| SOS148, SOS149, SOS151, SOS173, SOS404, SOS468 | 112-447 (446-470) | CBAnd1 (GI.4) | Seg08-12 (GI.3) | $1.341x10^{-28}$ | $3.518x10^{-13}$ | $1.393x10^{-29}$ | $1.711x10^{-8}$ | $3.928x10^{-8}$ | $1.351x10^{-3}$ | $1.238x10^{-12}$ |

[a] 99% confidence interval.

Figure. ML tree for the structural gene p16 (n=221 sequences; nucleotides 1-429; nucleotide substitution model K2+Γ4). Sequences obtained in this study appear in bold and * mark the recombinants at the p16/p23 boundary.

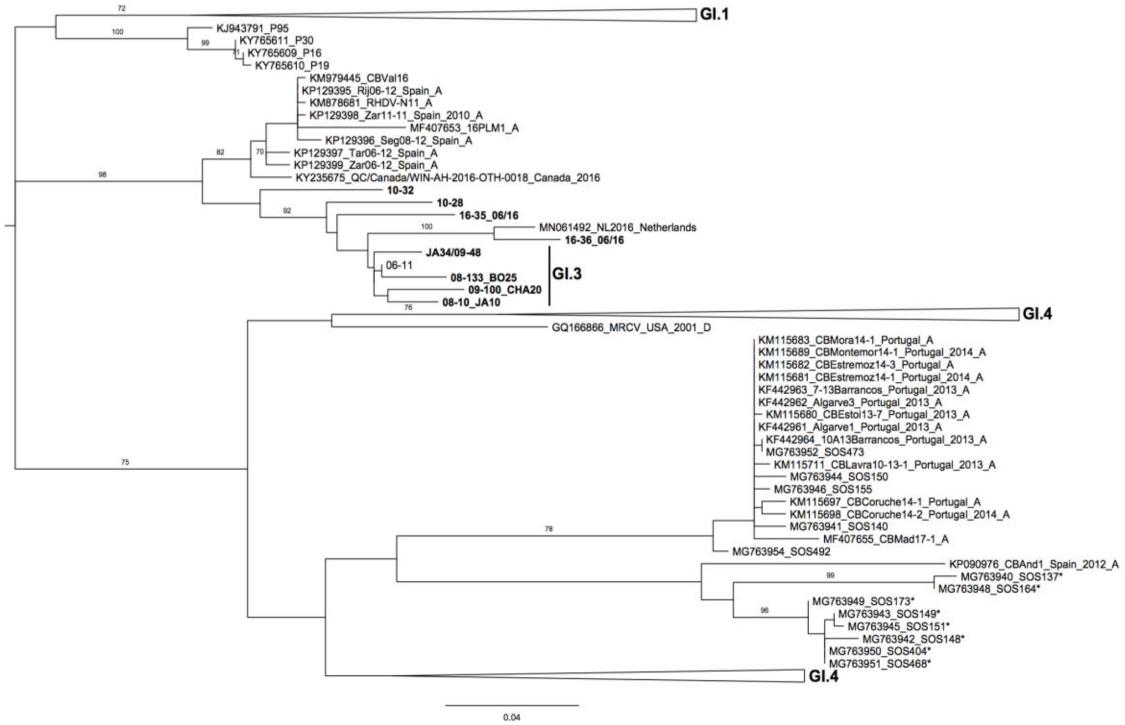

Table. Summary of the genomic composition of the GI.2 strains.

| Strains | Genomic partition | | |
|---|---|---|---|
| | **p16** | **NSP-p16*** | **SP**** |
| 10-28, 10-32, 16-35, 16-36 (France, 2010 and 2016); N11, Zar11-11, Tar06-12, Zar06-12, Rij06-12, Seg08-12 (Spain, 2011-2012); CBVal16 (Portugal, 2012); 16PLM1 (Canary Islands, Spain, 2016); RHDV2-NL2016 (Netherlands, 2016); QC/Canada/WIN-AH-2016-OTH-0018 (Canada, 2016) | GI.3 | GI.3 | GI.2 |
| CBAnd1 (Spain, 2012); Algarve1, Algarve3, 7-13_Barrancos, 10A13_Barrancos, CBEstoi13-7, CBEstremoz14-1, CBEstremoz14-3, CBMora14-1, CBMontemor14-1, CBCoruche14-1, CBCoruche14-2, CBLavra10-13-1, CBMad17-1, SOS140, SOS150, SOS155, SOS473, SOS492 (Portugal, 2013-2017) | GI.4 | GI.4 | GI.2 |
| 13-165 (France, 2013); CBMert14-1, CBMert14-2, CBAlgarve14-1, CBAlgarve14-3, CBAlgarve14-4, SOS089, SOS125, SOS129, SOS133, SOS158, SOS474, PSM2, CBPico17-1, CBPico17-2, CBMad17-2, CBMad17-3 (Portugal, 2014-2017); BLMT-1 (Australia, 2015); RED2016_Poland_Lodzkie_Set-2016, VMS_Poland_West Pomeranian_Jun-2017 (Poland 2016-2017) | GI.1b | GI.1b | GI.2 |
| SOS137, SOS164 (Portugal, 2015) | GI.4 | GI.1b | GI.2 |
| SOS148, SOS149, SOS151, SOS173, SOS404, SOS468 (Portugal, 2015) | GI.4 | GI.3 | GI.2 |
| AUS/NSW/2016_10_10/CAR-3 (Australia, 2016) | GI.1a | GI.1a | GI.2 |

* Non-structural proteins other than p16.

** Structural proteins.
